# Data on consistency among different methods to assess atherosclerotic plaque echogenicity on standard ultrasound and intraplaque neovascularization on contrast-enhanced ultrasound imaging in human carotid artery

**DOI:** 10.1016/j.dib.2016.09.041

**Published:** 2016-10-03

**Authors:** Mattia Cattaneo, Daniel Staub, Alessandra P. Porretta, Jeanne Marie Gallino, Paolo Santini, Costanzo Limoni, Rolf Wyttenbach, Augusto Gallino

**Affiliations:** a Hospital of San Giovanni, Department of Cardiology, Bellinzona, Switzerland; b University Hospital Basel, Department of Angiology, Basel, Switzerland; c Hospital of San Giovanni, Department of Radiology, Bellinzona, Switzerland; d University of Applied Sciences and Arts of Southern Switzerland, Switzerland

**Keywords:** Contrast enhanced ultrasound, Echogenicity, Carotid atherosclerosis

## Abstract

Here we provide the correlation among different carotid ultrasound (US) variables to assess echogenicity n standard carotid US and to assess intraplaque neovascularization on contrast enhanced US. We recruited 45 consecutive subjects with an asymptomatic≥50% carotid artery stenosis. Carotid plaque echogenicity at standard US was visually graded according to Gray–Weale classification (GW) and measured by the greyscale median (GSM), a semi-automated computerized measurement performed by Adobe Photoshop^®^. On CEUS imaging IPNV was graded according to the visual appearance of contrast within the plaque according to three different methods: CEUS_A (1=absent; 2=present); CEUS_B a three-point scale (increasing IPNV from 1 to 3); CEUS_C a four-point scale (increasing IPNV from 0 to 3). We have also implemented a new simple quantification method derived from region of interest (ROI) signal intensity ratio as assessed by QLAB software. Further information is available in “Contrast-enhanced ultrasound imaging of intraplaque neovascularization and its correlation to plaque echogenicity in human carotid arteries atherosclerosis (M. Cattaneo, D. Staub, A.P. Porretta, J.M. Gallino, P. Santini, C. Limoni et al., 2016) [1].

**Specifications Table**

**Table:** 

Subject area	*Medicine*
More specific subject area	*Peripheral artery imaging and atherosclerosis*
Type of data	*Tables*
How data was acquired	*Standard US ultrasound and contrast-enhanced ultrasound*
Data format	*Analyzed*
Experimental factors	*The data reports correlation coefficients and p-value between each plaque echogenicity variable and each contrast-enhanced ultrasound variable*
Experimental features	*Carotid artery standard duplex ultrasound examination was performed to obtain images adequate for greyscale median measurement, performed offline. Then, contrast-enhanced ultrasound was performed according to subsequent visual and quantitative analysis.*
Data source location	*Bellinzona − 6500, Switzerland*
Data accessibility	*Data is within this article*

**Value of the data**.

Consistency among results obtained by different methods to grade carotid plaque echogenicity and to visually assess the intraplaque neovascularization suggests that they may be interchangeable.

– This may help to choose a visual method to assess IPNV by CEUS, since more reproducible methods may be preferred for future studies on carotid plaque.
– This may also help to compare new quantitative methods to assess IPNV by CEUS (such as dynamic computer assisted analyses and 3D volume data sets), which are currently being developed.
– Moreover, we provide here a new potentially useful, simple method for quantification of IPNV on US. However, it should be validated by histology.

## 1 Data

The data represented are provided in tables that display the correlation among different carotid ultrasound (US) variables.

1. Correlation among echogenicity variables on standard carotid US as assess by the visual grading according Gray–Weale classification and measured by semi-automated according GSM-derived variables (assess by Adobe Photoshop^®^) (Table 1).
2. Correlation among different visual methods to assess intraplaque neovascularization on contrast enhanced US and a new simple quantification method derived from region of interest (ROI)signal intensity ratio as assessed by QLAB software (Philips Healthcare NV, Amsterdam, The Nederlands) (Table 2).

## 2 Experimental design, materials and methods

### 2.1 Study population

Patients were recruited from among consecutive subjects, who underwent carotid duplex ultrasound examinations. Inclusion and exclusion criteria have been previously published [1]. This is a sub-analysis of the same recruited patients (*n*=40), who have completed the US and CEUS examination. The study was approved by the local ethic review board and registered on clinicaltrial.gov (NCT02321410) [1].

### 2.2 US examination

The same experienced operator acquired all the extracranial cerebral arteries duplex ultrasound, by Philips iE33 xMatrix (Philips Healthcare NV, Amsterdam, The Nederlands) equipped with a 9 to 3 extended MHz linear array probes. The examination was implanted according to current guidelines as previously described [1].

#### 2.2.1 Standard duplex ultrasound and GSM

Importantly, to obtain images adequate for greyscale median (GSM) measurement, US scanning set was optimized to visualize the media-adventitia interface at the far wall, but to avoid noise in the vessel lumen. These scanner sets permit to obtain similar average greyscale levels of the regions lying deeply and superficially from the scanner point of view, allowing subsequent image normalization and plaque analysis. Standard US images were normalized and GMS was calculated by Adobe Photoshop CS6 software (Adobe System, San Jose, CA, USA) as previously published [1]. The following parameters were calculated on the grey-scale image: median value (GSM), first percentile value (percent_25), third percentile value (percent_75), the percent of pixel with greyscale value under 25 (pep25) and 32 (pep32) [2], [3]. Each lesion echogenicity was also graded according to GW visual classification as follows [4]: uniformly echolucent (class I), predominantly echolucent (class II), predominantly echogenic (class III), or uniformly echogenic or extensively calcified (class IV).

#### 2.2.2 Contrast enhanced Ultrasounds (CEUS)

CEUS was performed according to a previously described protocol [1], [5], [6] and following the US standard examination. The CEUS investigation focused on the single lesion per patient. US scanner settings used to acquire CEUS imaging has been previously published [1]. We performed CEUS imaging using sulphur hexafluoride-containing phospholipid microbubbles as a contrast agent (SonoVue; Bracco Spa, Milan, Italy) with a previously described protocol in order to minimize pseudo-enhancement artefacts [1]. A 30-second carotid CEUS loop-recording (CEUS-LR) was acquired and stored as DICOM image file for subsequent analysis. No adverse events occurred during the study.

### 2.3 CEUS grading

IPNV grading was performed according to the visual appearance of microspheres within the plaque profile as previously described in various studies [5], [6]. IPNV were identified by the dynamic movement of the echogenic reflectors (microspheres) observed within the hypoechoic plaque profile, which is in contrast with the highly-enhanced vessel lumen. We used three different visual methods to grade IPNV. Different working groups have previously published the following methods:

– CEUS_A [6]*:* Grade 1=no appearance of bubbles within the plaque or bubbles confined to plaque adventitial side; Grade 2=appearance of bubbles within the plaque moving from the adventitial side or shoulder reaching plaque core.
– CEUS_B [7]: Grade 1=no appearance of bubbles within the plaque; Grade 2=moderate appearance of bubbles within the plaque*;* Grade 3=extensive appearance of bubbles within the plaque.
– CEUS_C [5]: Grade 0=no appearance of neovascularization within the plaque; Grade 1=limited appearance of neovascularization within the plaque; Grade 2=moderate neovascularization within the plaque; Grade 3=presence of a pulsating, arterial vessel within the plaque.

IPNV was also measured using QLAB image-manager software (Philips Healthcare NV, Amsterdam, The Nederland). IPNV was quantified by the QLAB-derived region of interest (ROI) ratio on a single frame (QLAB_ratio). From the dynamic CEUS-LR evaluation, each investigator independently chose a single still frame considered to be the most representative according to the highest amount of visually-detected microbubbles within the plaque. Two independent operators (MC and DS) drew two regions of interest on each selected frame. The first ROI (ROI1) was drawn to delineate the plaque by smooth polygon dragged clicking various points on the plaque contour, while the second ROI (ROI2) was drawn by a standard 5×5 mm square in the carotid lumen adjacent to the plaque in the same still frame. The software automatically calculates video intensity as decibel (dB) units on the still image. ROI-ratio was determined by ratio between RO1 and ROI2 intensity, so that the higher the ROI-ratio, the higher the estimated IPNV plaque burden. The present method has not been validated by means of histology.

## 3 Statistical methods

All statistical analyses were performed with IBM-SPSS software Version 22.0 (IBM Corporation, Software Group, Somers, NY, USA). Continuous data are shown as mean and SD, categorical data as numbers and proportions. Correlations were assessed using the non-parametric Spearman׳s Rho correlation coefficient and the *χ*-square test. Statistical significance was declared if the rounded two-tailed *p*-value will be less than 0.05.

## Figures and Tables

**Table 1:** Consistency among plaque echogenicity variables. Correlation coefficients and *p*-value are reported for each comparison. Level of significance *p* 0.01.

	GW	GSM	GSM_mean	pep_25	pep_32	percent_25	percent_75
	CC (*p*-value)	CC (*p*-value)	CC (*p*-value)	CC (*p*-value)	CC (*p*-value)	CC (*p*-value)	CC (*p*-value)
GW	1.000						
GSM	−0.706 (0.000)	1.000					
GSM_mean	−0.724 (0.000)	0.952 (0.000)	1.000				
pep_25	0.569 (0.000)	−0.850 (0.000)	−0.775 (0.000)	1.000			
pep_32	0.593 (0.000)	−0.907 (0.000)	−0.831 (0.000)	0.983 (0.000)	1.000		
percent_25	−0.579 (0.000)	0.901 (0.000)	0.818 (0.000)	−0.960 (0.000)	−0.974 (0.000)	1.000	
percent_75	−0.684 (0.000)	0.934 (0.000)	0.965 (0.000)	−0.701 (0.000)	−0.767 (0.000)	0.747 (0.000)	1.000

**Table 2:** Consistency among CEUS scores and quantification. Correlation coefficients and *p*-value are reported for each correlation. Level of significance *p* 0.01.

	CEUS_A	CEUS_B	CEUS_C	QLAB_ratio
	CC (*p*-value)	CC (*p*-value)	CC (*p*-value)	CC (*p*-value)
CEUS_A	1.000			
CEUS_B	0.713 (.000)	1.000		
CEUS_C	0.628 (.000)	0.841 (.000)	1.000	
QLAB_ratio	0.124 (.452)	−0.055 (.740)	−0.073 (.657)	1.000
